# Successful Conservative Approach of Managing Spontaneous Pneumomediastinum in a Young Female: A Case Report and Literature Review

**DOI:** 10.7759/cureus.91564

**Published:** 2025-09-03

**Authors:** Mohamad M Assker, Ebraheim S Alyammahi, Yousef S Alabrach, Obaid Ullah Khan

**Affiliations:** 1 Radiology, Sheikh Khalifa Medical City, Abu Dhabi, ARE; 2 Internal Medicine, Zayed Military Hospital, Abu Dhabi, ARE; 3 Internal Medicine, Sheikh Khalifa Medical City, Abu Dhabi, ARE; 4 Pulmonology, Sheikh Khalifa Medical City, Abu Dhabi, ARE

**Keywords:** boerhaave syndrome, chest pain, endoscopy, esophageal perforation, spontaneous pneumomediastinum

## Abstract

Spontaneous pneumomediastinum (SPM) is defined as gas in the mediastinum in the absence of structural damage to the aerodigestive tract. It occurs secondary to the Macklin phenomenon, which is alveolar rupture due to hyperdistension. Although it has a benign nature, most patients get admitted to the hospital and undergo unnecessary investigations. A 20-year-old female presented to the emergency department with retrosternal chest pain. She had no preceding history of vomiting or alcohol intake. She was diagnosed with celiac disease by endoscopic biopsy eight months ago. The patient was vitally stable. Chest examination was unremarkable. Basic labs revealed no elevation in inflammatory markers. Chest X-ray revealed pneumomediastinum. A pulmonologist was consulted and advised for computed tomography (CT) thorax with contrast, which confirmed the presence of pneumomediastinum with no associated subcutaneous emphysema or pleural effusion. The patient was discharged home with close follow-up in the pulmonology clinic. No antibiotics or further esophageal evaluation were performed. Her final diagnosis was SPM, and she continued to do well on further visits. Although endoscopy is a well-known cause of both SPM due to simple reflexes to scope manoeuvres like cough reflex, and secondary complex pneumomediastinum due to esophageal perforation, the patient’s benign course is more in favour of SPM. An early pulmonology consultation resulted in the safe discharge of the patient, preventing unnecessary admission and investigations. This highlights the benign nature of the condition and the potential cost savings associated with prompt diagnosis and treatment.

## Introduction

Mediastinal emphysema, or more commonly referred to as spontaneous pneumomediastinum (SPM), is a sinister-sounding yet benign entity. It is defined as the presence of gas in the mediastinum and, for the most part, is considered a diagnosis of the young [1]. It can occur as a byproduct of gas leakage from any mediastinal structure communicating with the outside, namely the upper respiratory tract, gastrointestinal tract, or thoracic airways [2]. Usually, it is documented among three peaks across the population’s age demographics. The first peak occurs during the neonatal period [3], typically due to the established Macklin phenomenon of pulmonary air leak in newborns, where alveolar overdistension secondary to air distribution imbalance results in alveolar rupture [4]. The second peak occurs among toddlers, mostly explained by high rates of incidence of airway infections associated with recurring bouts of increased airway pressure during cough and infrequent pulmonary parenchymal necrosis [5]. The final and most relevant peak in our case occurs among tall, thin male adolescents [6].

Leaking air can find its way into subcutaneous tissues, causing subcutaneous emphysema [7]. Air dissection can also reach the pericardium, peritoneum, or pleural cavity, leading to complications pertaining to each of those anatomic positions [8,9]. A chest X-ray is usually sufficient for diagnosis if lucent streaking outlining mediastinal organs is present [10]. Chest CT may, however, be used in inconclusive studies to confirm suspicions. Management is usually conservative with rest and analgesia, especially in uncomplicated cases [11]. Patients on mechanical ventilation can benefit from simple pressure reduction, while those suffering complicated SPM require case-by-case evaluation and appropriate treatment.

## Case presentation

A 20-year-old female presented to the emergency department with retrosternal chest pain. She had no preceding history of vomiting, alcohol intake, or drug abuse. Her past medical history was positive for a celiac disease diagnosis made by endoscopic biopsy eight months ago. The patient was vitally stable on examination. Chest examination was clear. Basic labs revealed no leukocytosis or elevation in inflammatory markers. Urea and lytes were normal.

**Figure 1:**
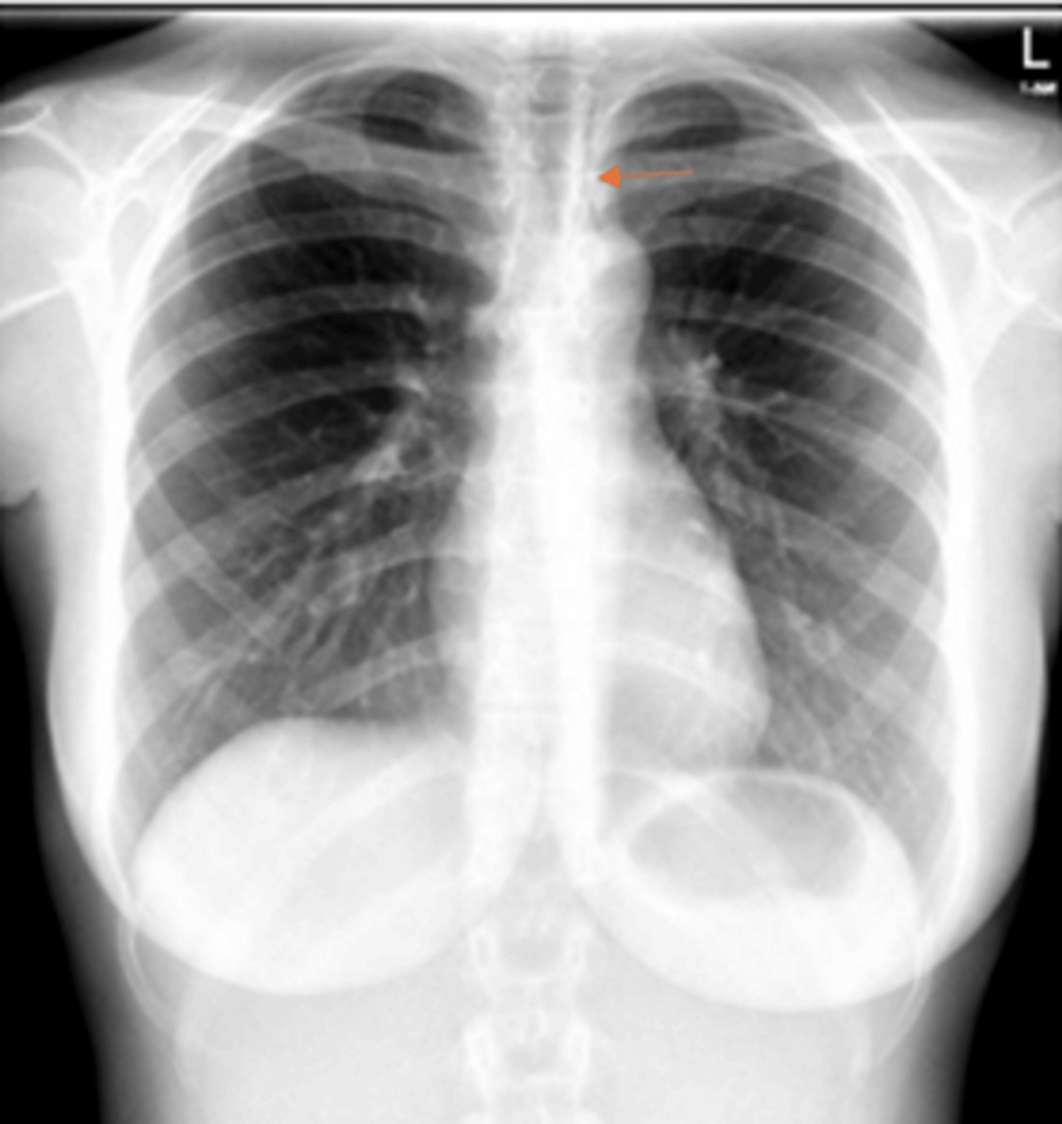
The air in the mediastinum can be best appreciated to the left of the trachea (orange arrow). No pleural effusion was seen on either side.

**Figure 2:**
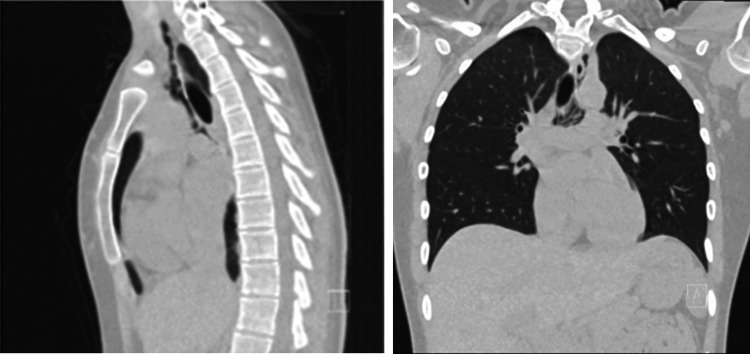
CT images confirm the presence of pneumomediastinum in sagittal (left panel) and coronal (right panel) views. No associated complications or pleural effusion are seen.

A chest X-ray was performed, which revealed pneumomediastinum with no pleural effusion (Figure 1). The pulmonologist was consulted, and he advised computed tomography (CT) thorax with contrast, which confirmed the presence of pneumomediastinum with no associated subcutaneous emphysema or pleural effusion (Figure 2). The patient was discharged home with close follow-up in the pulmonology clinic. No antibiotics or further esophageal evaluation were performed.

During clinic follow-up, the patient was doing well, with an improvement in her chest pain. Her final diagnosis is spontaneous pneumomediastinum. A repeat CXR, performed approximately nine days after the initial one, showed improvement in her pneumomediastinum, and she continued to do well on subsequent visits to other clinics.

## Discussion

Spontaneous pneumomediastinum (SPM) is a relatively rare condition with a reported prevalence of 0.002% [12]. It is not common in adults and predominantly affects young male individuals, with an average age of 23 years, and a male-to-female ratio of 3:1 [13]. In general, pneumomediastinum could be a simple and self-limited condition like SPM or could be a life-threatening complication of an underlying disease [14]. The etiology of pneumomediastinum can be divided into primary and secondary, where primary pneumomediastinum is the spontaneous pneumomediastinum with no history of trauma or iatrogenic cause [13,14]. Secondary pneumomediastinum can be related to esophageal rupture, previous trauma, or invasive procedure [13,14]. In primary pneumomediastinum, sudden intrathoracic pressure changes are highly associated with the disease [13]. Identified risk factors are smoking history, recreational drug inhalation, cough, asthma, physical exertion, retching, and emesis [12,13].

Considering this patient’s history of endoscopy, a presumed iatrogenic etiology of SPM was hypothesized. Endoscopy may be the culprit in developing either spontaneous or secondary complex pneumomediastinum, the former being caused by simple reflexes to scope maneuvers, such as cough and gag reflexes, while the latter is caused by perforating the esophagus [15]. The latter condition, also known as Boerhaave syndrome, can give a similar radiological appearance to SPM but is life-threatening. The prolonged and relatively benign course of her symptoms is more suggestive of primary SPM, which may spark the question of "How long a pneumomediastinum might persist before causing serious harm if unresolved on its own?" The natural course of SPM usually takes anywhere between two days and two weeks, with an initial worsening of chest pain and other symptoms that eventually resolve completely [16]. Recurring episodes are recorded in less than 5% of cases and may be another possibility behind this patient’s bizarre and remote presentation [17].

A myriad of SPM complications can be the reason behind the patient’s visit for such a benign condition. Pneumothorax secondary to SPM is highly unlikely in the absence of mechanical ventilation, especially with a chronic duration and lack of shortness of breath as presented [18]. This also applies to tension mediastinum, which is a life-threatening complication requiring ultrasound-guided needle drainage [19]. Pneumopericardium is commonly found with pneumomediastinum associated with COVID-19 [20].

## Conclusions

Spontaneous pneumomediastinum is a rare but benign entity that is mostly mistaken for a life-threatening condition such as Boerhaave syndrome. Early recognition and appropriate follow-up ensure safe patient disposition and avoid the unnecessary costs of intensive investigations usually performed for this condition.
